# Multiple *rpoB* Mutants of *Mycobacterium tuberculosis* and Second-order Selection

**DOI:** 10.3201/eid1007.030598

**Published:** 2004-07

**Authors:** Igor Mokrousov

**Affiliations:** * St. Petersburg Pasteur Institute, St. Petersburg, Russia

**Keywords:** letter, *Mycobacterium tuberculosis*, hypermutability, *rpoB*, rifampin resistance

**To the Editor:** Rad and colleagues recently described variation in some genes involved in DNA repair (*mutT2*, *mutT4*, *ogt*) in *Mycobacterium tuberculosis* strains of different genotypes (1). This approach can also be used to investigate developing rifampin resistance in the context of emerging mutator alleles. Resistance to rifampin in *M. tuberculosis* strains is usually caused by the point mutations in the *rpoB* gene encoding the β-subunit of the DNA-dependent RNA polymerase, which is a target of the drug. Although a single point mutation is sufficient for developing rifampin resistance, a number of articles (2,3) describe multiple *rpoB* mutants for *M. tuberculosis*, i.e., rifampin-resistant strains harboring mutations in different codons of *rpoB*. Double, triple, and quadruple mutations in *M. tuberculosis* clinical isolates were reported in studies conducted throughout the world (2,3). Such emergence, albeit infrequent, of *M. tuberculosis rpoB* multiple mutants raises questions about their biologic importance and underlying mechanisms; answers to both remain elusive.

I propose an explanation of these observations in terms of second-order selection of hypermutable (mutator) alleles based on alterations in DNA repair genes. Unlike that of other antituberculosis drugs, resistance to rifampin is acquired in most *M. tuberculosis* isolates by altering a single target molecule and offers the most appropriate and straightforward model to demonstrate possible hypermutability in this species. In mycobacteria, hypermutability was demonstrated in vitro for *M. smegmatis*, a surrogate model for *M. tuberculosis*, as an increase in reversion (mutant to wild-type) rate in *rpoB526* or *rpsL43* under counterselection by streptomycin or rifampin, respectively (4). A correlation between high mutation rate and antimicrobial resistance was reported for *Pseudomonas aeruginosa* isolates from lungs of cystic fibrosis patients (5). The mutator *P. aeruginosa* strains resulted from a defective mismatch-repair system (5). In *M. tuberculosis*, mismatch-repair genes (*mutH*, *mutL*, *mutS*, and *recJ*) were not found in its genome (6). However, the nucleotide pool in this species is exceptionally clean because of the presence of several copies of the *mutT* gene (1,6); the MutT protein removes oxidized guanines (8-Oxo-dGTP), thus counteracting replication or transcription errors. Consequently, the MutHLS mismatch-repair system simply may be not required in *M. tuberculosis* (6). Therefore, hypermutability in some strains of this species resulting in multiple *rpoB* mutants might develop under certain special (in vivo) circumstances through inactivation or down-regulation of some *mutT* genes. Further, the two most frequently described *rpoB* mutations are 531TCG→TTG and 526CAC→TAC. Both are cytosine-to-tymine transitions, which easily occur by spontaneous cytosine deamination to uracil. Indeed, *M. tuberculosis* is a G+C rich organism, therefore, it is naturally at high risk for cytosine deamination. Furthermore, pathogenic mycobacteria are at increased risk for deamination because of the production of reactive oxygen and nitrogen intermediates inside host macrophages. This deamination process is normally counteracted by uracil-N-glycosylase, the product of the *ung* gene, and organisms defective in the removal of uracil from DNA have an increased spontaneous mutation rate and more G:C→ A:T base-pair transitions (7). Merchant et al., by using *ung*+ and *ung*– *Escherichia coli* strains, demonstrated that total nitric oxide exposures in the mmol/L range can lead to C→T mutations by a mechanism probably involving cytosine deamination (8). On the other hand, in *M. smegmatis*, the abrogation of the Ung activity leads not only to increased mutator phenotype but also to growth inhibition by reactive nitrogen intermediates (7). In summary, I speculate that mutations in *ung* that do not completely impair function, but do decrease synthesis of its product, might tolerably increase the spontaneous C→T mutations, including those in the respective positions in the *rpoB* codons 531 and 526. This assumption seems likely because both of the aforementioned particular mutations were described in spontaneous mutants of H37Rv obtained in vitro and had a Darwinian fitness slightly less than or equal to that of the *rpoB* wild-type-susceptible parental strain (9). In contrast, the translesion synthesis-based pathways appear less likely to contribute to emergence of such mutants, although at least one of the translesion synthesis genes (*dinP*) is present in the genome of *M. tuberculosis*. In the *E. coli* in vitro model, a translesion synthesis enzyme (*dinB* encoded DNA polymerase IV) activity clearly promoted more important frameshift mutations (single-base deletions) in two thirds of the spontaneous mutants (10).

From an evolutionary point of view, the multiple *rpoB* mutations in *M. tuberculosis* have been hypothesized to arise as a compensatory mechanism to ameliorate the fitness costs of the original resistance mutation by a secondary mutation (11). The process of adaptation to the fitness costs of chromosomally encoded resistance has been studied in *E. coli* and *Salmonella enterica* serovar Typhi for mutations that affect translation in the *rpsL* and *fusR* genes (11) and for *rpoB* mutations in *E. coli* K12 strain (11). In the last instance, the *rpoB* multiple mutants were selected in vitro in a stepwise fashion, and one double mutant, L511Q+D516G (also described in *M. tuberculosis* strain [3]), exhibited a relative fitness either greater than or equal to either single mutant or the wild type. Reynolds (11) suggested that this allele is favored not merely as a combination of two low-level resistance mutations but also because these mutations together boost resistance and preserve fitness. Whether the same is true for other multiple mutant alleles in *M. tuberculosis rpoB* remains to be seen. Studying the costs of resistance of multiple *rpoB* mutations in a more realistic environment of animal models of TB infection seems promising.
